# Whole genome sequence analysis of the first reported isolate of *Salmonella* Agona carrying blaCTX-M-55 gene in Brazil

**DOI:** 10.1038/s41598-023-29599-5

**Published:** 2023-02-09

**Authors:** Amanda Maria de Jesus Bertani, Thais Vieira, Alex Domingos Reis, Carla Adriana dos Santos, Elisabete Aparecida de Almeida, Carlos Henrique Camargo, Monique Ribeiro Tiba Casas

**Affiliations:** Present Address: Bacteriology Division, Instituto Adolfo Lutz, 351, Doutor Arnaldo Avenue, São Paulo, Brazil

**Keywords:** Microbiology, Antimicrobials

## Abstract

This study analyzes the genomic findings of the first report of *Salmonella* isolate carrying the *bla*_CTX-M-55_ gene, recovered from a bacteremic patient from Brazil. A bacterial isolate positive for the *bla*_CTX-M-55_ gene was submitted to antimicrobial susceptibility testing by disk diffusion and epsilometric test. Whole genome sequencing was performed using Illumina technology. Conjugation assay was performed; plasmid sizes determined by S1-PFGE and plasmid content were investigated by hybrid assembly after MinION long reads sequencing. Isolate 288_18 was identified as sequence type ST13, resistant to ampicillin, cefotaxime, ceftazidime, cefepime, ceftriaxone, and aztreonam. A transferable IncFII plasmid sized approximately 67 kb was found to carry the *bla*_TEM-1_ and *bla*_CTX-M-55_ in a module consisting of IS26*-bla*_TEM-1B_*-*WbuC*-bla*_CTX-M-55_-IS26. In addition, an 117 kb IncI1plasmid was also identified in the 288_18 isolate, but without additional resistance genes. To the best of our knowledge, this is the first report of *bla*_CTX-M-55_ in *Salmonella* isolated from human infection in Brazil. The occurrence of *bla*_CTX-M-55_ in the IncFII epidemic plasmid in a relevant clinical human isolate of *Salmonella* Agona underscores the urgent need for enhanced and effective continuous surveillance for controlling its dissemination.

## Introduction

Salmonellosis is one of the most common food-borne human diseases worldwide^1^. Within the last years, *Salmonella enterica* subsp. *enterica* serovar Agona has been one of the top 20 most commonly reported serotypes causing human infections^2^. Although cases of gastroenteritis due to *Salmonella enterica* usually resolve without medical intervention, antimicrobial therapy is recommended for patients with severe disease^3^. In these cases, antibiotics are used to treat invasive nontyphoidal *Salmonella,* like fluoroquinolone, cephalosporin, and azithromycin^4^. Currently, multidrug-resistant *S*. Agona poses a significant hazard to human and animal health, given that the emergence of *Salmonella* isolates resistant to extended-spectrum cephalosporins (ESCs) is a worldwide public health concern^5^. Infection with extended-spectrum beta-lactamases (ESBL)-producing organisms is particularly concerning since few treatment options exist and worse clinical outcomes can occur.

Plasmid-mediated ESBL plays a vital role in the increasing frequency of multidrug-resistant Enterobacterales, among which CTX-M-type are the most common ESBL type found in recent years^6^. CTX-M-55 is a variant of CTX-M-15, containing a single amino acid substitution (Val77Ala) that contributes to enhanced cephalosporin-hydrolyzing activity^6^. This variant has been reported in several countries. In China, CTX-M-55 is the second most common CTX-M ESBL, and identical plasmids have been observed in humans and animals, indicating their spread in different reservoirs^7,8^. CTX-M-55 was detected to rapidly increase in prevalence, especially in *Escherichia coli* from animals^9^.

The first report on CTX-M-55-producing *Salmonella* in human isolates came from the United States and China in 2011^10,11^. This enzyme has also been reported in different serotypes of *Salmonella* in Europe, Asia, China, and Thailand^12–14^, but there is no report of CTX-M-55 in *Salmonella* isolates from Brazil. In this study, we describe the genomic findings of the first report of a *Salmonella isolate* carrying the *bla*_CTX-M-55_ gene recovered from a bacteremic patient from Brazil.

## Materials and methods

### Bacterial isolate

Adolfo Lutz Institute is a regional reference laboratory for *Salmonella* isolates from São Paulo, Brazil. Thus, all isolates received are submitted to classical serotyping, antimicrobial susceptibility testing, and molecular-typing by PFGE and next-generation sequencing. Isolate 288_18 was recovered from a blood sample from a patient with clinical symptoms of bacteremia in 2018.

### Serotyping and antimicrobial susceptibility testing

The serotype was determined according to the Kauffmann–LeMinor scheme^15^. Antimicrobial susceptibility testing was performed by the disk-diffusion method according to the guidelines and interpretation criteria of the CLSI^16^ (nalidixic acid, amoxicillin/clavulanic acid, ampicillin, amikacin, aztreonam, ceftazidime, cefotaxime, ceftriaxone, cefepime, ciprofloxacin, chloramphenicol, streptomycin, gentamicin, imipenem, trimethoprim-sulfamethoxazole, sulfonamide, and tetracycline). Gradient diffusion was performed to nalidixic acid, ciprofloxacin, ceftriaxone, and cefotaxime, and broth microdilution to colistin, and polymyxin B according to CLSI guidelines^16^.

### Conjugation

Conjugation experiments were conducted in Luria–Bertani (LB) broth using sodium azide-resistant *E. coli* J53 as the recipient strain. Transconjugants were selected on MacConkey agar containing ceftriaxone (2.5 µg/mL) and sodium azide (100 µg/mL). In addition, an antimicrobial susceptibility test was performed to confirm the antimicrobial resistance characteristics of these transconjugants. The donor and the respective transconjugant isolates were submitted to S1-PFGE to identify the size of large plasmids carrying the *bla*_CTX-M-55_ gene.

### Whole-genome sequencing and analysis

Whole-genome shotgun sequencing was performed using the Illumina MiSeq (Illumina, San Diego, CA, USA). For DNA extraction, strains were grown on Luria Bertani Agar overnight at 37 °C. Subsequently, a single colony was inoculated in 2 mL of Luria–Bertani broth for 12 h at 37 °C. The suspension was used for bacterial cultures to continue extraction and purification by the Wizard Genomic DNA Purification kit (Promega, USA). High-quality DNA (assessed by gel electrophoresis and Qubit quantification) was submitted to library preparation with Illumina DNA Prep Tagmentation and sequenced in a MiSeq (USA) instrument with MiSeq Reagent Kit v3 (150 cycles). Quality control was performed with FastQC and Kraken2 software, within the Galaxy Europe Server (https://usegalaxy.eu).

Long-read sequencing and base-calling of select isolates were performed using Oxford Nanopore technology (Oxford, United Kingdom). Libraries were constructed using a rapid multiplex barcoding kit. Sequencing was performed using an Oxford Nanopore MinION device with R9.4.1 flow cells. Base-calling and read processing was performed using Guppy v2.3.1 using default parameters. The quality of the raw sequence reads was checked by the interactive program FastQC. The genome was assembled using a combination of short and long reads by the Unicycler 0.4.8 hybrid assembler. Genomes were automatically annotated with Prokka v1.14.6. The tools mentioned above were used within the Galaxy Europe Server (https://usegalaxy.eu). Annotations and alignments were visualized in the Artemis Comparison Tool (ACT)^17^. Online Center for Genomic Epidemiology tools was used to determine in silico serotype and MLST, to detect acquired resistance genes and chromosomal mutations associated with antimicrobial resistance, and for plasmid classification (PlasmidFinder, PBRT, pMLST; https://cge.cbs.dtu.dk/services/). Blast software compared similar plasmids following alignment using BioNumerics v.8.1 (bioMérieux/Belgium). Software BLAST Ring Image Generator (BRIG)^18^ was used to compare sequence of plasmid 288_18 with other representative plasmids to further generate circular plasmid maps. Core genome MLST (cgMLST) was performed in BioNumerics version 8.1 (bioMérieux/Belgium) using the *S.enterica* cgMLST scheme of 3002 target loci available on Enterobase (https://enterobase.warwick.ac.uk/). The comparison was performed using the 175 genomic sequences of *Salmonella* Agona deposited in the Enterobase and PubMLST database (https://pubmlst.org/). We searched for isolates with “ST13” listed as MLST in the strain metadata and “Agona” as the serovar in either the strain metadata or experimental data. The complete nucleotide sequence of strain 288_18 has been deposited into the GenBank database under the accession number: JAODHS000000000.

## Results

Strain 288_18 was isolated from the blood of a 73-year-old patient and identified as *Salmonella enterica* subsp. *enterica* serovar Agona, sequence type ST13. The phenotypic test (disk diffusion) revealed resistance to ampicillin, cefotaxime, ceftazidime, cefepime, ceftriaxone, and aztreonam. The isolate was susceptible to the remaining antimicrobials tested. The double disk synergism test was positive for ESBL production. Minimal inhibitory concentration (MIC) showed that isolate 288_18 was sensitive to nalidixic acid (6 µg/mL), and ciprofloxacin (0.015 µg/mL). Still, it confirmed resistance in high levels to ceftriaxone (32 µg/mL), cefotaxime (256 µg/mL), cefepime (16 µg/mL), and aztreonam (64 µg/mL).

**Figure 1:**
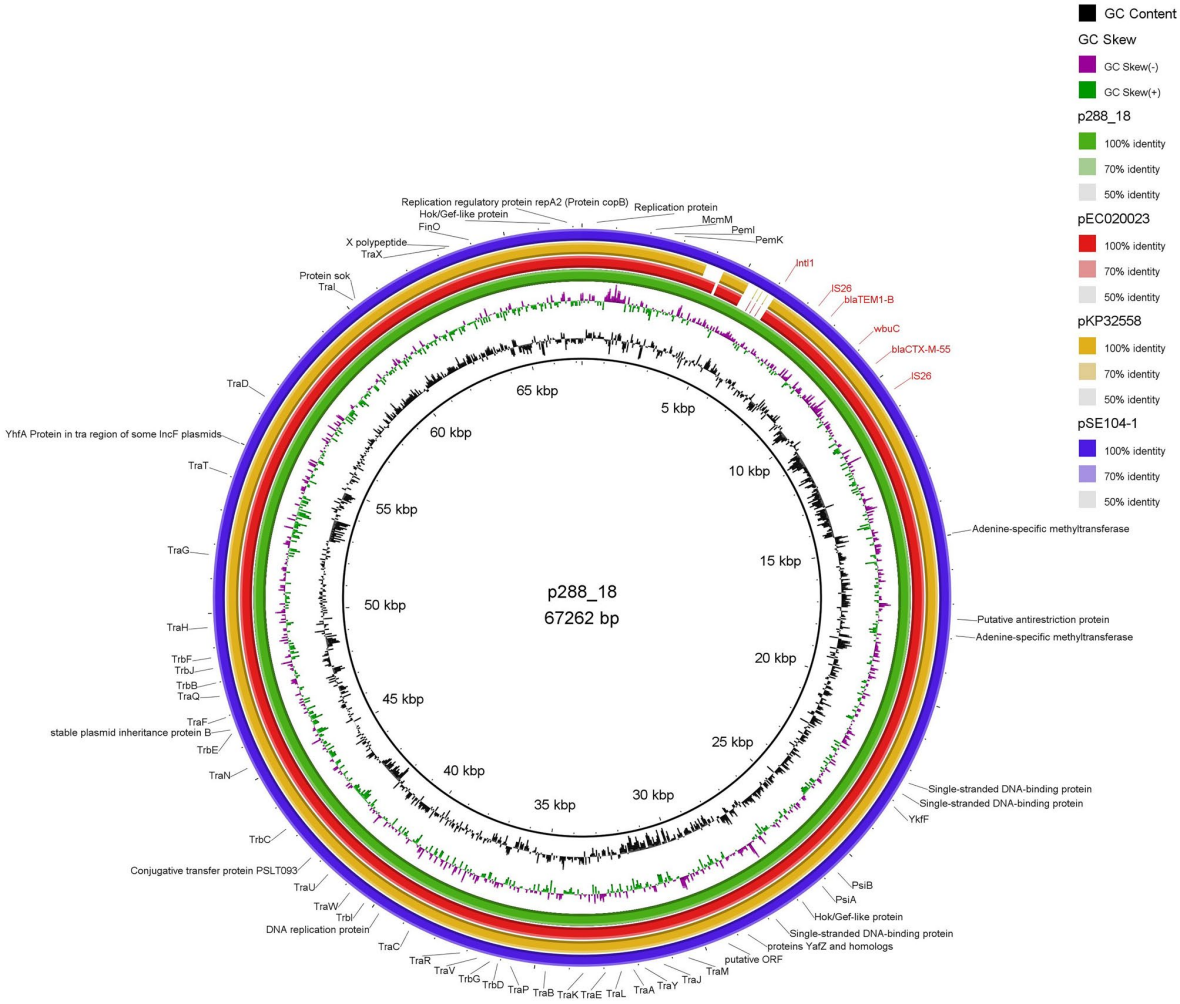
BRIG (Blast Ring Image generator) comparison of the p288_18 of IncFII- type *bla*_CTX-M-55_*-*carrying plasmid against the two IncFII plasmids (accession numbers: CP025949.1, CP076034.1, CP050713.1). P288_18 containing the *bla*_CTX-M-55_ gene was used as a reference. Each ring corresponds to a plasmid (indicated at the right of the figure together with the color code). Circle 1 (innermost) displayed the scale in kilobase pairs. Circle 2 and 3 displayed the GC content and GC skew, respectively. Regions of the plasmid are labelled on the outer circle.

A transferable IncFII plasmid, pMLST F33:A-:B-, sized approximately 67 kb, was found to carry the *bla*_TEM-1B_ and *bla*_CTX-M-55_*,* in a module consisting of IS26*- bla*_TEM-1B_*-*WbuC*-bla*_CTX-M-55_-IS26, associated with phenotypic resistances detected to ampicillin, amoxicillin/clavulanate, cefotaxime, ceftazidime, ceftriaxone, and cefepime*.* Further comparative analysis of the complete plasmid indicated that *bla*_CTX-M-55_ was inserted in an IncFII plasmid in a nearly identical manner to *E.coli* strain SCEC020023 plasmid pEC020023-FII (GenBank Accession nº CP025949.1), *K. pneumoniae* strain KP32558 plasmid pKP32558-4-FII (GenBank Accession nº CP076034.1) and *S*. Enteritidis strain SE104 plasmid pSE104-FII (GenBank accession nº CP050713.1) (Fig. 1). These three plasmids were identified in Chinese isolates recovered from urine, bronchoalveolar lavage fluid, and stool, respectively, all of them also carrying the *bla*_CTX-M-55_ and *bla*_TEM-1B_ genes.

Conjugative transfer of *bla*_CTX-M-55_ was successfully achieved to *E. coli* J53 as the recipient. The ceftriaxone and cefotaxime MIC values of the transconjugant strain remained the same observed for the wild strain, ceftriaxone (32 µg/mL) and cefotaxime (256 µg/mL). S1-PFGE identified two plasmids compatible with hybrid assemblies generated with short and long reads.

**Figure 2:**
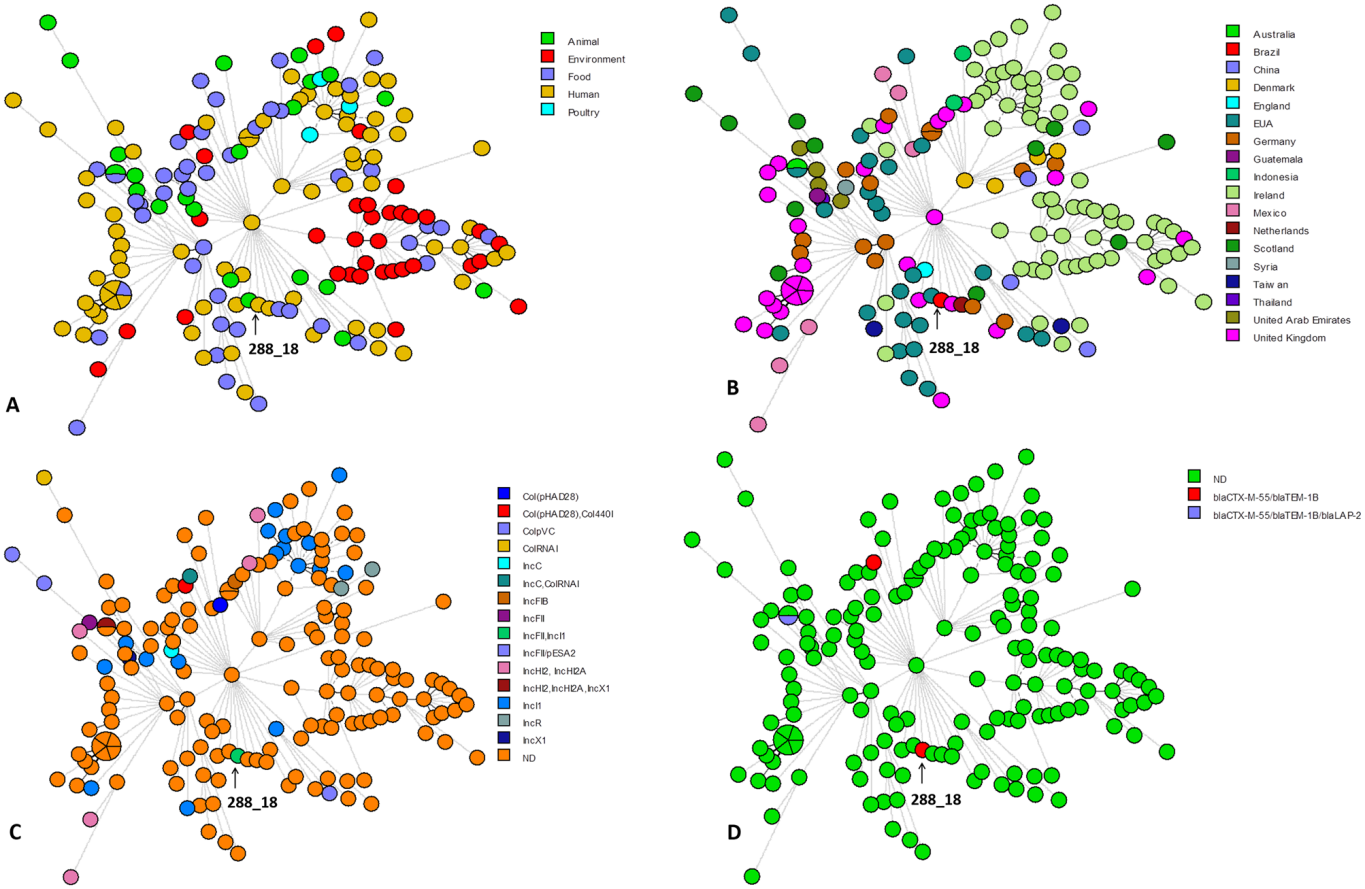
A Minimum-spanning tree based on cgMLST analysis of 175 *Salmonella* Agona strains (Supplementary Table 1). The figure was generated using BioNumerics version 8.1 (bioMérieux/Belgium). The entire set of genomes and all its metadata and genotyping results are available to the registered users of PubMLST at (https://pubmlst.org/) and Enterobase (https://enterobase.warwick.ac.uk/). (**A**) Isolates are color-coded by their source of origin as shown in the legend. (**B**) Clinical isolate (288_18) was grouped within a clade formed with ST13 *S*. Agona isolates related to strains of human, animal and food origin from different countries and it was linked with an isolate from the human from United Kingdom with 27 alleles distance. (**C**) Isolates are color-coded by their Inc type plasmids as shown in the legend. (**D**) Isolates are color-coded by the presence and ∕or absence of ESBL genes as shown in the legend. ND (not detected).

In addition, an 117 kb IncI1 plasmid (ST 231) was also identified in the 288_18 isolate, but without additional resistance genes. The chromosome *fosA7* and *aac(6′)-Iaa* genes were found, which can confer resistance to fosfomycin and aminoglycosides, respectively. Still, the phenotypic resistance test was sensitive to these drug classes. The minimum spanning tree showed that isolate 288_18 presented the highest similarity (27 alleles distance) with an epidemiologically unrelated isolate recovered from a human source in the United Kingdom, dated 1972 (Fig. 2; Supplementary Table 1).

## Discussion

To the best of our knowledge, this is the first report of *bla*_CTX-M-55_ in *Salmonella* isolated from human infection in Brazil. *Salmonella* Agona is a serovar is increasingly recognized as a cause of foodborne disease outbreaks and has the potential to both be multidrug resistant and cause disease^2^. The *bla*_CTX-M-55_ gene has already been found in *S*. Agona in Australia isolated in silver gull^3^, but it was not localized in an epidemic plasmid like IncF. Wild birds, implicated as vectors of antimicrobial resistance internationally, are colonized by MDR and virulent lineages of *Enterobacteriaceae* that threatent to human health^3^.

The IncFII plasmid identified in this study was found to be disseminated in other *Salmonella* serotypes, indicating a potential well-adapted plasmid spreading several antimicrobial resistance genes in different reservoirs^19,20^. IncF-like plasmids represent the main mechanisms by which epidemic clones can arise^3^. In Brazil, the *bla*_CTX-M-55_ gene is circulating into a lineage of IncFII plasmids especially in *E. coli* from animal origin^21^. In *Salmonella*, the *bla*_*CTM-55*_ gene is mainly found in IncHI2^22^, IncI2, and IncF replicon-type plasmids^19,23^. Occurrence of *bla*_CTX-M-55_ was also reported to exist in the bacterial chromosome^24^. The identification of the *bla*_CTX-M-55_ gene in a genetic environment surrounded by IS26 in an IncFII plasmid represents potential clinical, animal and environment issues and need to be monitored.

The phylogenetic analysis showed that isolate 288_18 presented the highest similarity with a strain from the human isolated in the United Kingdom. The occurrence of *bla*_CTX-M-55_ in the IncFII epidemic plasmid in a relevant clinical human isolate of *Salmonella* Agona underscores the urgent need for enhanced and effective continuous surveillance for controlling its dissemination.

## Conclusions

The antimicrobial surveillance of *Salmonella* strains using whole genome sequencing allowed the first identification of *bla*_CTX-M-55_, which confers resistance to third-generation cephalosporin in Brazil. *Salmonella* Agona is a serovar is increasingly recognized as a cause of foodborne disease outbreaks and has the potential to be both multidrug-resistant and disease-causing. The occurrence of the ESBL gene in an epidemic plasmid reinforces the need for enhanced continuous surveillance of antimicrobial resistance in isolates of both human and non-human sources and alerts to prevent possible failures in the antimicrobial treatment of severe invasive salmonellosis.

## Supplementary Information

Supplementary Table 1.

## Data Availability

Accession numbers: This Whole Genome Shotgun project has been deposited at DDBJ/ENA/GenBank under the accession JAODHS000000000. The version described in this paper is version JAODHS010000000.
